# Complete Genome Sequence of *Bacillus thuringiensis* subsp. *thuringiensis* Strain IS5056, an Isolate Highly Toxic to *Trichoplusia ni*

**DOI:** 10.1128/genomeA.00108-13

**Published:** 2013-03-21

**Authors:** Emilia Murawska, Krzysztof Fiedoruk, Dennis K. Bideshi, Izabela Swiecicka

**Affiliations:** a Department of Microbiology, Institute of Biology, University of Bialystok, Bialystok, Poland; b Department of Microbiology, Medical University of Bialystok, Bialystok, Poland; c Department of Entomology, University of California, Riverside, Riverside, California, USA; d Department of Natural and Mathematical Science, California Baptist University, Riverside, California, USA

## Abstract

The genome sequence of the entomopathogen *Bacillus thuringiensis* subsp. *thuringiensis* strain IS5056 was determined. The chromosome is composed of 5,491,935 bp. In addition, IS5056 harbors 14 plasmids ranging from 6,880 to 328,151 bp, four of which contain nine insecticidal protein genes, *cry1Aa3*, *cry1Ab21*, *cry1Ba1*, *cry1Ia14*, *cry2Aa9*, *cry2Ab1*, *vip1*, *vip2*, and *vip3Aa10*.

## GENOME ANNOUNCEMENT

Various strains of *Bacillus thuringiensis* are used worldwide as natural biopesticides to control insect pests. Their entomo-pathogenicity is due to insecticidal proteins produced during vegetative growth (vegetative insecticidal proteins [Vips]) and sporulation (crystal δ-endotoxins [Cry]; cytolytic toxins [Cyt]) (1). *B. thuringiensis* subsp. *thuringiensis* strain IS5056 (serotype H1), isolated in 2005 from soil collected in Biebrza National Park (Poland), produces a quasicuboidal bipyramidal crystal composed of Cry toxins highly toxic to *Trichoplusia ni* larvae (2) and also harbors *vip* genes (3) encoding toxins known to enhance activity of Cry proteins (4).

The total genomic DNA of IS5056 was used to construct three libraries: (i) a GS FLX+ shotgun library using the GS FLX+ library preparation kit, (ii) an 8-kb-long paired-end library using the GS FLX paired-end kit, and (iii) an Illumina paired-end library using the Illumina TruSeq2.0 kit (Roche Diagnostics GmbH, Mannheim, Germany). The libraries were sequenced using the genome sequencer FLX+ System (Roche), which yielded 178 million nucleotides covering the genome ~18-fold, and the Illumina HiScanSQ genome analyzer (Illumina Inc.), which generated ~400 million nucleotides covering the genome ~40-fold. All high-quality reads were assembled into 161 contigs in 21 scaffolds with the Newbler *de novo* assembler (454 sequencing system software; Roche). Gaps were filled using the Expand long-template PCR system (Roche), after which the PCR amplicons were sequenced in the ABI3500 genetic analyzer (Applied Biosystems).

The 6.8-Mb genome of IS5056 consisted of a 5,491,935-bp circular chromosome and 14 circular replicons: pIS56-6 (6,880 bp), pIS56-8 (8,251 bp), pIS56-9 (9,671 bp), pIS56-11 (11,331 bp), pIS56-15 (15,185 bp), pIS56-16 (16,206 bp), pIS56-39 (39,749 bp), pIS56-63 (63,864 bp), pIS56-68 (68,616 bp), pIS56-85 (85,134 bp), pIS56-107 (107,431 bp), pIS56-233 (233,730 bp), pIS56-285 (285,459 bp), and pIS56-328 (328,151 bp) (Table 1). The G+C contents of these replicons ranged from 31.0% to 35.7% for pIS56-107 and pIS56-15, respectively, and did not deviate significantly from that of the chromosome (35.4%). Annotation of protein-coding genes (CDSs) was performed using the RAST system (5). The IS5056 chromosome harbors 5,617 CDSs, 85 tRNAs, and 13 rRNA operons. Plasmids represent ~19.0% of the total CDSs. The four megaplasmids, pIS56-328, pIS56-285, pIS56-233, and pIS56-107, contain 302, 294, 186, and 130 CDSs, respectively. The remaining 10 plasmids, pIS56-85, pIS56-68, pIS56-63, pIS56-39, pIS56-16, pIS56-15, pIS56-11, pIS56-9, pIS56-8, and pIS56-6, contain 111, 89, 61, 49, 18, 16, 23, 7, 13, and 6 CDSs, respectively.

**Table 1:** The sequence features of 14 plasmids from *Bacillus thuringiensis* subsp. *thuringiensis* strain IS5056

Plasmid	Length (bp)	G+C content (%)	No. of CDSs (forward/reverse)	Total length of CDSs (bp)	Coding sequences (%)^*a*^	Average length of CDSs (range) (bp)	GenBank accession no.
pIS56-328	328,151	32.6	302 (144/158)	237,030	72.2	784.9 (114-8,583)	CP004137
pIS56-285	285,459	33.0	294 (163/131)	206,469	72.3	702.3 (114-4,020)	CP004136
pIS56-233	233,730	32.7	186 (71/115)	179,304	76.7	964.0 (114-10,011)	CP004135
pIS56-107	107,431	31.0	130 (93/37)	89,415	83.2	687.8 (114–3,687)	CP004134
pIS56-85	85,134	33.2	111 (35/76)	65,664	77.1	591.6 (117–3,435)	CP004133
pIS56-68	68,616	31.8	89 (17/72)	56,721	82.7	637.3 (114–2,655)	CP004132
pIS56-63	63,864	34.7	61 (12/49)	55,212	86.5	905.1 (132–3,594)	CP004131
pIS56-39	39,749	34.9	49 (17/32)	33,015	83.1	673.8 (120–3,024)	CP004130
pIS56-16	16,206	33.3	18 (11/7)	10,497	64.8	583.2 (141–2,139)	CP004129
pIS56-15	15,185	35.7	6 (3/13)	10,557	69.5	659.8 (123–2,964)	CP004128
pIS56-11	11,331	31.6	23 (16/7)	8,850	78.1	384.8 (114–1,380)	CP004127
pIS56-9	9,671	33.0	7 (0/7)	5,559	57.5	794.1 (294–1,338)	CP004126
pIS56-8	8,251	32.4	13 (5/8)	5,292	64.1	407.1 (123–1,230)	CP004125
pIS56-6	6,880	31.8	6 (2/4)	3,189	46.4	531.5 (141–1,095)	CP004124

a Ratio of total gene length to plasmid length.

Altogether, IS5056 harbors nine genes encoding insecticidal proteins, which reside on four plasmids. The *vip1* and *vip2* genes occur in pIS56-328. The *cry1Aa3*, *cry1Ia14*, *cry2Aa9*, and *cry2Ab1* homologues, together with the *vip3Aa10* gene, create a pathogenicity island in pIS56-285. The *cry1Ba1* and *cry1Ab21* homologues are present in pIS56-107 and pIS56-63, respectively. The availability of the IS5056 genome should facilitate the study of plasmid and insecticidal protein gene diversity and the regulation of insecticidal protein expression and will contribute to deeper understanding of the evolution of entomopathogenicity among *B. thuringiensis* strains.

### Nucleotide sequence accession numbers.

The complete genome sequence of *B. thuringiensis* IS5056 has been deposited in GenBank under accession numbers CP004123 (chromosome) and CP004124 to CP004137 (plasmids).
